# Intratumoral immunotherapy with the TLR7/8 agonist 3M-052

**DOI:** 10.1186/2051-1426-1-S1-P138

**Published:** 2013-11-07

**Authors:** DV Smirnov, G Zhao, JM Beaurline, KE Johnson, JT Capecchi, LI Harrison, MA Tomai, JM Elvecrog, DM Klinman, JP Vasilakos

**Affiliations:** 1 Drug Delivery Systems Division, 3M Company, St. Paul, MN, USA; 2 Center for Cancer Research, National Cancer Institute, Frederick, MD, USA

## 

TLR agonists have demonstrated anti-tumor activity in both animals and humans. Clinically, successful anti-tumor activity with TLR agonists was most often observed when the molecules were applied locally to dermatological cancers. This mode of administration was also associated with fewer and less severe adverse events (AEs) when compared to systemic administration. The efficacy of TLR agonists in human cancer patients has been limited at best, in part reflecting the dose-limiting toxicity associated with systemic administration of this form of therapy. Moreover, recent nonclinical studies indicate that intratumoral administration of TLR agonists helps reverse the immunosuppressive microenvironment that protects large established tumors from immune elimination. In light of these findings, we developed a new class of lipophilic TLR7/8 agonists that are ideal for intratumoral injection. The molecules are retained at the site of injection, thereby supporting the focused induction of anti-tumor immunity. As release into the systemic circulation is low, we postulate that the generation of AE-inducing systemic cytokines is low. Results from studies in mice and rats using 3M-052 confirms that this lipophilic TLR 7/8 agonist is retained at the injection site. Direct activation of innate immune cells with anti-tumor activity was observed, as was a reduction in the immunosuppressive local environment. 3M-052 also induces the accumulation of mononuclear phagocytes concomitant with the induction of histologically established tumor liquifactive necrosis. Moreover, 3M-052 induces antitumor activity when injected into tumors but not when injected distal from tumors. These findings are based on inhibition of tumor growth and increased long-term survival. 3M-052 is under consideration for clinical use in patients with cancers suitable for intratumoral therapy and as being evaluated as a cancer vaccine adjuvant.

